# Effects of heme oxygenase-1 on innate and adaptive immune responses promoting pregnancy success and allograft tolerance

**DOI:** 10.3389/fphar.2014.00288

**Published:** 2015-01-06

**Authors:** Anne Schumacher, Ana C. Zenclussen

**Affiliations:** Department of Experimental Obstetrics and Gynecology, Medical Faculty, Otto-von-Guericke UniversityMagdeburg, Germany

**Keywords:** allo-antigens, allograft, heme oxygenase-1, immune tolerance, pregnancy

## Abstract

The heme-degrading enzyme heme oxygenase-1 (HO-1) has cytoprotective, antioxidant, and anti-inflammatory properties. Moreover, HO-1 is reportedly involved in suppressing destructive immune responses associated with inflammation, autoimmune diseases, and allograft rejection. During pregnancy, maternal tolerance to foreign fetal antigens is a prerequisite for successful embryo implantation and fetal development. Here, HO-1 has been implicated in counteracting the overwhelming inflammatory immune responses towards fetal allo-antigens, thereby contributing to fetal acceptance. Accordingly, HO-1 ablation negatively impacts the critical steps of pregnancy such as fertilization, implantation, placentation, and fetal growth. In the present review, we summarize recent data on the immune modulatory capacity of HO-1 towards allo-antigens expressed by the semi-allogeneic fetus and organ allografts. In this regard, HO-1 has been shown to promote alloantigen tolerance by blocking dendritic cell maturation resulting in reduced T cell responses and increased numbers of regulatory T cells. Moreover, HO-1 is suggested to shift the uterine cytokine milieu towards a protective Th2 profile and protects fetal tissue from apoptosis by upregulating anti-apoptotic molecules. Thus, HO-1 is not only a pivotal regulator of the initial steps of pregnancy; but also, an important player in supporting the maternal immune system in tolerating the fetus.

## INTRODUCTION

Heme oxygenase-1 (HO-1) has been widely described as an enzyme with anti-inflammatory, antioxidant, and cytoprotective functions. Moreover, due to its ability to modulate immune responses HO-1 has become an interesting target in several clinical specialties including infectious diseases, immunology (autoimmune diseases), oncology, transplantation, and obstetrics (fetal tolerance). HO-1 catalyzes the degradation of heme into biliverdin, carbon monoxide (CO) and free iron, and there is evidence that HO-1 mediates its functions through these byproducts. In response to inflammatory stimuli and oxidative stress HO-1 is induced in most cell types (Chauveau et al., 2005) where its expression activates or suppresses cell-intrinsic pathways. Hence, HO-1 is considered as one of the key regulators in the immunological network. Here, we focus this review on the immune regulating properties of HO-1 towards allo-antigens presented by the semi-allogeneic fetus or organ allografts on a cell-specific basis.

Under normal circumstances, recognition of foreign antigens by allo-reactive T cells causes inflammatory processes that result in the destruction of foreign antigens. During pregnancy, it is known that some degree of inflammation is required to ensure successful implantation of the blastocyst into the maternal endometrium. However, pregnancy complications may develop if inflammation becomes too excessive and is not counterbalanced by specific tolerance against paternal antigens. An adequate regulation of inflammation is thus a prerequisite for normal fetal development, and we believe that HO-1 is crucially involved in this process. In normal pregnancies, two HO isoforms, HO-1 and HO-2, are present in human, rat and mouse placenta (Kreiser et al., 2003; McLaughlin et al., 2003; Watanabe et al., 2004). HO-1 and HO-2 are differentially expressed and localized in the human placenta (McLean et al., 2000; Yoshiki et al., 2000). Extensive work from our group revealed that HO-1 controls critical phases throughout pregnancy including ovulation, fertilization, placentation, and fetal growth (Zenclussen et al., 2011, 2012; Linzke et al., 2014). The role of HO-1 in pregnancy was also explored by other research groups (Ahmed et al., 2000; Denschlag et al., 2004; Zhao et al., 2008,^2009^). Absence of HO-1 expression and/or its activity is associated with pregnancy complications in humans (Zenclussen et al., 2003; Denschlag et al., 2004) and mice (Zenclussen et al., 2011). Pharmacologic or gene therapy-mediated HO-1 upregulation protects the fetus from rejection (Sollwedel et al., 2005; Zenclussen et al., 2006) revealing the therapeutic potential of HO-1 in the treatment of pregnancy disorders.

In contrary to the physiologic condition of pregnancy, where the maternal immune system is naturally challenged by fetal allo-antigens, allotransplantation can be seen as an artificial situation where the immune system of the recipient must tolerate the foreign antigens expressed by the graft against its nature to accept it. However, it can be assumed that similar mechanisms contribute to allo-tolerance in both conditions and the knowledge of basic mechanisms by which the fetus guarantees its own survival may help to improve allograft acceptance. In agreement with observations made for pregnancy, HO-1 augmentation before or after allotransplantation is associated with increased allograft survival (Soares et al., 1998; DeBruyne et al., 2000; Chen et al., 2010; Evans et al., 2012). Thus, modulating the HO-1 system may be a promising tool to prevent acute and chronic rejections of allotransplants.

## EFFECTS OF HO-1 ON MONOCYTES AND MACROPHAGES

Numbers, subtypes, and functions of monocytes and macrophages change during normal pregnancy. Both immune cell populations favor blastocyst implantation and placentation by promoting trophoblast invasion, spiral artery remodeling, and angiogenesis [recently reviewed in (Faas et al., 2014)]. Through its metabolite CO, HO-1 provokes differentiation of progenitors into functional macrophages. Hence, ablation of HO-1 has been shown to prevent macrophage differentiation and expression of its markers (Wegiel et al., 2014). Accordingly, a lack of HO-1 or its byproducts may impair implantation and placentation by affecting macrophage generation and activity.

Interestingly, HO-1 may also alter the course of autoimmune diseases and infections in pregnancy via modulation of myeloid-derived immune cell populations including monocytes and macrophages. Multiple sclerosis (MS), a Th1-dominated autoimmune disease, significantly improves during the third trimester of pregnancy and this is thought to be due to pregnancy-specific alterations of immune responses. In a well-established mouse model for MS, the experimental autoimmune encephalomyelitis (EAE), the authors observed that animals having a myeloid-specific HO-1 deficiency showed persistent activation of antigen-presenting cells (APCs), enhanced Th17 infiltration, and increased myelin-specific T cell reactivity resulting in a higher disease activity (Tzima et al., 2009). Moreover, Chora et al. (2007) nicely demonstrated that HO-1 and CO reduced disease activity in EAE mice. Although highly speculative, it can be assumed that HO-1 induction in pregnancy is involved in the attenuation of MS symptoms during this time. However, at present there is no direct proof for this beneficial effect.

Bacterial infections occurring during pregnancy are often associated with spontaneous abortions or preterm births. Macrophages are directly activated by microbial products and are involved in the first line of defense against bacterial invasion and colonization. Lipopolysaccharide (LPS), a component of the outer membrane of gram-negative bacteria, has been shown to induce HO-1 upregulation in rodent macrophages (Immenschuh et al., 1999), followed by a decreased expression of several pro-inflammatory genes (Otterbein et al., 2000). Additionally, gram-negative bacteria, such as *Helicobacter pylori*, induce polarization of macrophages to a more regulatory phenotype, resulting in a dampening of anti-bacterial immune responses (Gobert et al., 2014). Moreover, cell specific HO-1 inhibition in rodent macrophages was associated with a reduced expression of IFN-β after infection with the gram-positive bacteria *Listeria monocytogenes*. Because *Listeria* infection depends on IFN-β production, HO-1-deficient animals showed enhanced bacterial clearance and survival, while control mice succumbed to infection (Tzima et al., 2009). On the other hand, Tachibana et al. (2011) showed that *Listeria* infections decrease HO-1 expression and induce placental cell death. This is in agreement with our observations that mice infected with *Tritrichomonas foetus* lost their concepti and displayed a lower HO-1 expression in the uterus (Woudwyk et al., 2012). These findings suggest that regulation of HO-1 levels by bacteria favor bacterial persistence and, thereby facilitate embryonic death.

In organ transplantation, the effect of HO-1 on macrophages or its expression by these cells is proposed to favor allograft tolerance. Pre-treatment of pancreatic allografts prior to transplantation with cobalt protoporphyrin (CoPP), a HO-1 inducer, markedly increased HO-1 expression in donor macrophages. This was associated with a significant decrease in inflammatory cytokines and an increase in anti-inflammatory cytokines to result in adequate graft function and survival (Becker et al., 2007). In agreement, pre-treatment of recipients or islet allografts with bilirubin, a reduction product of biliverdin, before transplantation improved graft survival in recipient mice. Moreover, donor treatment only upregulated HO-1 mRNA expression and reduced the number of macrophages that infiltrate islet grafts in recipient mice (Wang et al., 2006).

## EFFECTS OF HO-1 ON GRANULOCYTES

The effect of HO-1 on granulocytes in pregnancy and in allotransplantation has not been well studied. However, studies performed in rodent models of inflammation showed that HO-1 and its metabolites CO and biliverdin are able to reduce leukocyte rolling, adhesion, and neutrophil infiltration as well as migration of eosinophils to inflammatory sites (Freitas et al., 2006; Xia et al., 2007; Lin et al., 2010; Chiang et al., 2013; Zhang et al., 2013). In a human study, it was reported that treatment of partially purified basophils with hemin, a HO substrate analog, significantly induced biliverdin production in these cells, indicating that they do express HO. Additionally, hemin and exogenous CO seem to impair basophil activation (Vannacci et al., 2003). Interestingly, one study showed that activated regulatory T cells (Treg) initiate HO-1 expression in neutrophils rendering them to a suppressive phenotype (Lewkowicz et al., 2013). As Treg are major players in the establishment and maintenance of allo-tolerance, HO-1 induction in neutrophils may represent one mechanism by which Treg regulate innate immune responses towards allo-antigens.

## EFFECTS OF HO-1 ON MAST CELLS

Mast cells (MCs) are key regulators of allergic diseases. After activation, MCs release pre-formed and newly synthesized mediators to induce inflammatory immune responses. Several studies have suggested a protective role of HO-1 and its metabolites in allergic inflammation partially mediated through MCs. Induction of HO-1 expression in MCs suppressed their degranulation and production of inflammatory cytokines (Takamiya et al., 2002; Yasui et al., 2007; Pae et al., 2008; Matsushima et al., 2009). Furthermore, HO-1-expressing murine MCs restrained dendritic cell (DC) maturation *in vitro* followed by a decreased capacity of the DCs to provoke proliferation of spleen mononuclear cells (Ma et al., 2014).

Recently, we uncovered a critical role of MCs in pregnancy success. We found that uterine MCs (uMCs) display a unique phenotype and increase in number when a female becomes receptive (Woidacki et al., 2013). Additionally, we confirmed an increase in HO-1 levels at the same time (Zenclussen et al., 2014). Hence, it is conceivable that HO-1 is involved in uterine homing of MCs as well as in their local regulation. However, a direct impact of HO-1 on MC migration and functionality needs to be confirmed. After pregnancy occurs, uMCs rapidly expand and contribute to trophoblast survival, placentation, and fetal growth (Woidacki et al., 2013) – processes in which HO-1 plays major roles (Zenclussen et al., 2006, 2011, 2012). It is, however, to remark that an exaggerated augmentation of MC numbers or their activation can be associated with negative pregnancy outcomes [reviewed in (Woidacki et al., 2014)]. Further studies are needed to analyze whether there is a direct association between HO-1 and MCs in pregnancy.

The role of MCs in allograft rejection or tolerance is still not defined. There is evidence that inflammation resulting in allograft rejection is suppressed by a reciprocal relationship between MCs and Treg. While MCs contribute to Treg migration and function in the graft, Treg were shown to mitigate MC degranulation. However, in the case of allergy, intragraft or systemic MC degranulation may impair Treg homing and suppression and may result in a breakdown of allograft tolerance (de Vries et al., 2009). The interaction between MCs and Treg in allograft tolerance is further underscored by a study of Lu et al. (2006). The authors showed that activated Treg produce high levels of IL-9, thereby recruiting and activating MCs in tolerant allografts where MCs can mediate regional immune suppression (Lu et al., 2006). To what extent HO-1 can regulate MC function and thereby contribute towards MC-mediated allograft acceptance is not fully understood and requires further investigations.

## EFFECTS OF HO-1 ON NATURAL KILLER CELLS

Natural killer (NK) cells are the cytotoxic lymphocytes of the innate immune system that mainly respond to virally infected cells and tumor cells. During pregnancy, a unique subtype of NK cells, the so-called uterine NK (uNK) cells, represent the major cell fraction in the uterus. uNK cells express a specific receptor repertoire that differs from that of peripheral NK cells. However, the origin of uNK cells is still not defined. They may be converted from peripheral NK cells that are recruited to the uterus or may expand via *in situ* proliferation (Inoue et al., 1996; Borzychowski et al., 2003; van den Heuvel et al., 2005). In contrast to their undefined origin, there is a general consensus that they have an indispensable role for appropriate spiral artery remodeling that guarantees sufficient fetal nutrition (Hatta et al., 2011). We recently showed that HO-1 expression is important for the presence of uNKs and their expansion during pregnancy. We found that HO-1-deficient implantation sites have low uNK cell numbers and a decreased expression of uNK-related angiogenic factors. This phenotype was associated with shallow spiral artery formation leading to intrauterine growth restriction (IUGR) and gestational hypertension. Interestingly, we were able to restore the phenotype by application of low dose of CO during early to mid-gestation. CO treatment provoked an *in situ* proliferation of uNK cells, normalizing their numbers and further modulating angiogenic parameters that finally favor vascular remodeling and the prevention of hypertension (Linzke et al., 2014). Our results are in agreement with a study by Zhao et al. (2011) who showed that a partial HO-1 deficiency led to insufficient spiral artery remodeling and alterations of uNK differentiation and maturation. The authors further revealed that maternal HO-1 levels, but not fetal HO-1 levels, are important for a successful formation of the fetomaternal interface. These findings suggest a major influence of HO-1 on uNK cells during normal pregnancy (Zhao et al., 2011). Besides its pregnancy-favoring effect on uNK cells, HO-1 may also regulate NK cell function in allotransplantations, supporting allograft acceptance. It is well accepted that NK cells can promote rejection of allogeneic hematopoietic stem cells (Cudkowicz and Rossi, 1972). Furthermore, NK cells may also initiate solid organ transplant rejection by activating detrimental adaptive immune responses. In this regard, NK cells have been shown to eliminate immature DCs while sparing mature DCs, a behavior that promotes increased T cell activation (Degli-Esposti and Smyth, 2005). Moreover, NK cells can directly interact with T cells and induce their differentiation into Th1 cells (Martin-Fontecha et al., 2004). By killing Treg, NK cells further drive T cell-dependent allograft rejection (Roy et al., 2008). Application of heme into rats preceding cardiac allotransplantation augmented HO-1 levels followed by a reduction of peripheral NK cell numbers and prolonged allograft survival (Shen et al., 2011). However, data confirming a protective effect of HO-1 on NK cell-mediated allograft rejection are still scarce and further studies are still needed to clarify this point.

## EFFECTS OF HO-1 ON DENDRITIC CELLS

Dendritric cells can be seen as intermediates between the innate and adaptive immune systems and have a major impact on the fate of the semi-allogeneic fetus and the allograft. By presenting allo-antigens, mature DCs can induce the activation and clonal expansion of allo-reactive T cells, thereby fostering the rejection process. However, immature DCs, secreting mainly anti-inflammatory cytokines, have been reported to suppress allo-responses and may therefore play a rather favorable role in both pregnancy and transplantation.

In terms of an effect mediated by HO-1, DCs are one of the best-studied immune cell populations. Several studies indicate that HO-1 affects both phenotype and function of DCs. Spontaneous HO-1 expression could be confirmed in immature human and rat DCs, while maturation resulted in a drastic decline of HO-1. In line, induction of HO-1 by CoPP-inhibited LPS-induced DC maturation, secretion of pro-inflammatory cytokines, and induction of reactive oxygen species (ROS). Additionally, such HO-1-producing DCs retained their ability to produce IL-10 and had a lower T cell stimulatory capacity. Accordingly, inhibition of HO activity led to increased ROS levels, a mature phenotype, impaired phagocytic and endocytic functions, and increased T cell stimulatory capacity (Al-Huseini et al., 2014). These findings support the idea that HO-1 directs DCs towards a tolerogenic profile. Normal pregnancy is characterized by uterine DCs (uDCs) presenting an immature phenotype and mainly producing IL-10 (Kammerer et al., 2003; Blois et al., 2004). In contrast, abortions in mice are associated with an increased number of mature, IL-12-producing DCs (Blois et al., 2005). Our working group showed in collaboration with Dr. Tadokoro that, *in vivo*, DCs accumulate even before pregnancy during the estrous cycle, suggesting a role for these cells in embryo implantation (Zenclussen et al., 2013). Indeed, Plaks et al. (2008) revealed that a depletion of uDCs had detrimental effects on the decidualization process by interfering with the implantation of the embryos, which finally lead to their demise. Furthermore, HO-1 supports pregnancy success by retaining DCs in an immature state, thereby attenuating allo-reactive T cell responses and promoting the expansion of pregnancy-favoring Treg (Schumacher et al., 2012).

Similar to fetal rejection, failure of allograft acceptance is often caused by direct and indirect antigen-presenting pathways where DCs initiate anti-alloantigen-specific adaptive immune responses. In a murine model for transplant arteriosclerosis, adoptive transfer of HO-1-deficient DCs before allograft transplantation or inhibition of endogenous HO-1 in allograft recipients was associated with pronounced intragraft T cell infiltration and increased IgG deposition, resulting in elevated graft immunogenicity (Cheng et al., 2010). On the other hand, HO-1 induction in organ donors prior to organ transplantation may reduce activation of donor DCs and intra-allograft T cell infiltration while simultaneously promoting Treg development (Kotsch et al., 2007; Sun et al., 2011). These findings indicate that HO-1 expression has a major impact on DCs, an immune cell population with the ability to induce or suppress allo-responses.

## EFFECTS OF HO-1 ON B CELLS

B cells are allocated to the adaptive arm of the immune system and are best known for their capability to secrete antibodies. However, B cells are also able to efficiently present antigens and modulate the function of T cells and DCs by producing cytokines (Porakishvili et al., 2001; Youinou et al., 2005). Hence, it can be assumed that B cells are involved in essential immune regulatory processes promoting either fetal acceptance or rejection. We reported that various B cell subsets differentially affect pregnancy outcomes (Muzzio et al., 2013). In this regard, human B-1a B cells, known to produce poly reactive natural antibodies were reported to play a role in pre-eclampsia (Vannacci et al., 2003). In mice, B-1a B cells direct naive T cells towards an inflammatory Th1 and Th17 phenotype, and are therefore involved in cellular immune responses associated with pregnancy complications (Muzzio et al., 2014). In contrast, B cells contribute to fetal tolerance as well. It has been shown that the production of a structural unique type of antibodies, the so-called asymmetric antibodies, favor pregnancy success (Margni et al., 1976). Furthermore, there is evidence that IL-10-producing B cells with regulatory properties support fetal survival (Jensen et al., 2013; Rolle et al., 2013). The role of B cells in pregnancy has been recently reviewed (Fettke et al., 2014).

In the field of transplantation, B cells account for acute and chronic rejections (recently reviewed by (Nouel et al., 2014)). By presenting allo-antigens to T cells and by producing donor-specific antibodies, B cells initiate allograft rejection (Noorchashm et al., 2006). Intra-allograft B cell infiltration has therefore been associated with poor graft survival (Hippen et al., 2005). On the contrary, regulatory B cells (Bregs) have a rather favorable influence on allograft survival. Two human studies showed that an increased frequency of Bregs was found in tolerant renal transplanted patients (Newell et al., 2010; Pallier et al., 2010).

An action of HO-1 on B cells during pregnancy and organ transplantation is widely unexplored. HO-1 is expressed in normal and malignant human B cells after induction of oxidative stress, whereby malignant B cells exhibit a greater HO-1 expression (Bancos et al., 2010). Kapturczak et al. (2004) performed an elegant study, which compared the immune phenotype of HO-1 deficient with wild-type mice. Although the lack of HO-1 was associated with splenomegaly, analysis of splenocytes revealed no differences in the proportion of B cells. However, the authors found lower numbers of B220^+^ B cells in lymph nodes and significantly elevated serum IgM levels in HO-1-deficient animals (Kapturczak et al., 2004). In agreement, addition of heme to primary B cell cultures skews plasma cell differentiation toward the IgM isotype, decreasing IgG levels *in vitro* (Watanabe-Matsui et al., 2011). From these data, it can be assumed that in the presence of HO-1, destructive allo-antibody responses are controlled. In addition, injection of a recombinant adeno-associated viral vector (serotype 1) encoding IL-10 (rAAV1-IL-10) in a rat model of aortic allograft transplantation resulted in a significant reduction of graft infiltration with macrophages and B and T cells. The authors found that the protective effects of IL-10 were mediated through HO-1. This implies that HO-1 may regulate B cell infiltration into tissues where allo-antigens provoke inflammatory responses (Chen et al., 2005). This is a very interesting theory that needs further exploration.

## EFFECTS OF HO-1 ON T CELLS

T lymphocytes are key regulators of humoral- and cell-mediated immune responses. T helper cells expressing CD4 and CD8-expressing cytotoxic T cells are the two main T cell subpopulations. Furthermore, although oversimplified, T cells are usually subdivided into Th1, Th2, Th9, and Th17 cells according to their cytokine secretion pattern. By secreting cytokines, T cells define their local environment as either pro-inflammatory or anti-inflammatory. Normal pregnancy is characterized by a pro-inflammatory Th1 profile at early and late pregnancy stages that is critical for proper trophoblast invasion and later on for initiation of labor. At mid-gestation, mechanisms allowing the acceptance of fetal allo-antigens provoke a more Th2-dominated profile. Imbalances in these cytokine profiles are thus often associated with pregnancy complications (Hudic and Fatusic, 2009).

After organ transplantation allo-antigens trigger the activation and proliferation of T cell responses. Consequently, CD8^+^ T cells induce cell-mediated cytotoxicity, while CD4^+^ T cells promote B cell maturation and differentiation into allo-antibody-producing plasma cells. Additionally, T cells secrete soluble factors that activate and recruit immune cells to the site of the allograft and finally provoke its rejection.

Modulation of T cell responses is suggested to be one of the major mechanisms by which HO-1 contributes to fetal and allograft survival. During normal pregnancy, HO-1 expression may at least partially be responsible for the Th2 shift observed during mid-gestation. This is supported in our studies, which showed that adenoviral-mediated upregulation of HO-1 in abortion-prone animals increased the Th2/Th1 ratio and protected fetuses from rejection (Zenclussen et al., 2006). Accordingly, induction of HO-1 by CoPP in allotransplant recipients led to a reduction in the pro-inflammatory cytokine milieu (Ewing et al., 2007), leading us to propose a similar protective mechanism during allotransplantation.

Treg are central players in the regulation of fetal and allograft tolerances. While an augmentation of Treg at the fetomaternal interface and in the allograft is associated with pregnancy success and a good prognosis of graft acceptance, the lack of Treg often results in pregnancy complications and allograft rejection.

Several studies indicate that HO-1 and its byproducts affect the development and function of human and murine CD4^+^ and CD8^+^ Treg as well as the production of Treg-related suppressive molecules (Pae et al., 2003; Choi et al., 2005; El Andaloussi and Lesniak, 2007; Lee et al., 2007; Xia et al., 2007; Brandsma et al., 2008; Mougiakakos et al., 2011; Sun et al., 2011; Karimi et al., 2012). Andersen et al. (2009) propose that Treg expressing HO-1 are able to inhibit cytokine release, proliferation, and cytotoxicity of other immune cells. However, this is in contrast, to a study by Biburger et al. (2010) who reported that HO-1 expression by human Treg influences their proliferative behavior, but not their suppressive capacity. Moreover, Zelenay et al. (2007) provided evidence that in HO-1-deficient animals, Treg development, maintenance, and function are independent of HO activity. In these animals, the absence of HO-1 in APCs seemed to abolish the suppressive activity of Treg (George et al., 2008). After previously reporting that adoptive Treg transfer can rescue fetuses from immunological rejection (Schumacher et al., 2012), we investigated the influence of HO-1 on Treg-mediated fetal protection. We proved that inhibition of HO-1 abrogated the protective effects of the Treg transfer suggesting that HO-1 is involved in a Treg-mediated suppression during murine pregnancies (Schumacher et al., 2012). Altogether these findings suggest that HO-1 affects several important aspects in T cell biology, including T cell proliferation, cytokine secretion, and the induction of T cells with regulatory properties. Hence, modulation of T cell and in particular Treg responses through HO-1 represent a promising tool for the induction of fetal and allotransplant tolerance.

## EFFECTS OF HO-1 ON PROCESSES ENSURING TROPHOBLAST SURVIVAL

Placental development is a well-defined process of trophoblast proliferation and apoptosis. Irregularities within this process result in abnormal placentation and may cause pre-eclampsia, where an increased apoptotic rate of trophoblast cells was documented (Allaire et al., 2000; Sharp et al., 2010). HO-1 seems to directly influence trophoblast survival. By using a rat trophoblastic stem cell line that is capable of differentiating into trophoblast giant cells, we showed that HO-1 inhibition diminished cell viability and ability to differentiate into giant cells (Zenclussen et al., 2011). Thus, HO-1 regulates both the survival and maturation of trophoblast cells. Moreover, we found that adenoviral-mediated HO-1 induction in mice led to increased levels of the anti-apoptotic molecule Bag-1 and reduced the number of apoptotic cells in placental tissue (Zenclussen et al., 2006). In line, we recently confirmed that in partially deficient HO-1 animals, LPS treatment significantly reduced Bag-1 levels in placental tissue. In the same study, we found that human trophoblasts from spontaneous abortion patients had lower basal levels of HO-1 when compared to trophoblasts from normal pregnant women. The low HO-1 levels in trophoblasts from spontaneous abortion patients were further associated with a diminished Bag-1 expression (Kahlo et al., 2013). In an *in vivo* setting, we proved that exposure of CO during the implantation window and early placentation also reduced the number of apoptotic cells in the murine placenta and increased Bag-1 expression, implicating that the protective function of HO-1 might be mediated through its byproduct CO (El-Mousleh et al., 2012). Our work is in agreement with work from Zhao et al. (2009) who revealed that placentas from breedings of partially deficient HO-1 mice remained comparatively smaller and displayed lower placental weights than those from wild-type breedings. Lower HO-1 expression in heterozygous placentas was associated with an increase in apoptosis. These findings suggest that HO-1 not only modulates immune responses to ensure survival of fetal tissue; but also, supports pregnancy success by directly influencing trophoblast survival.

## CONCLUSION

**FIGURE 1:**
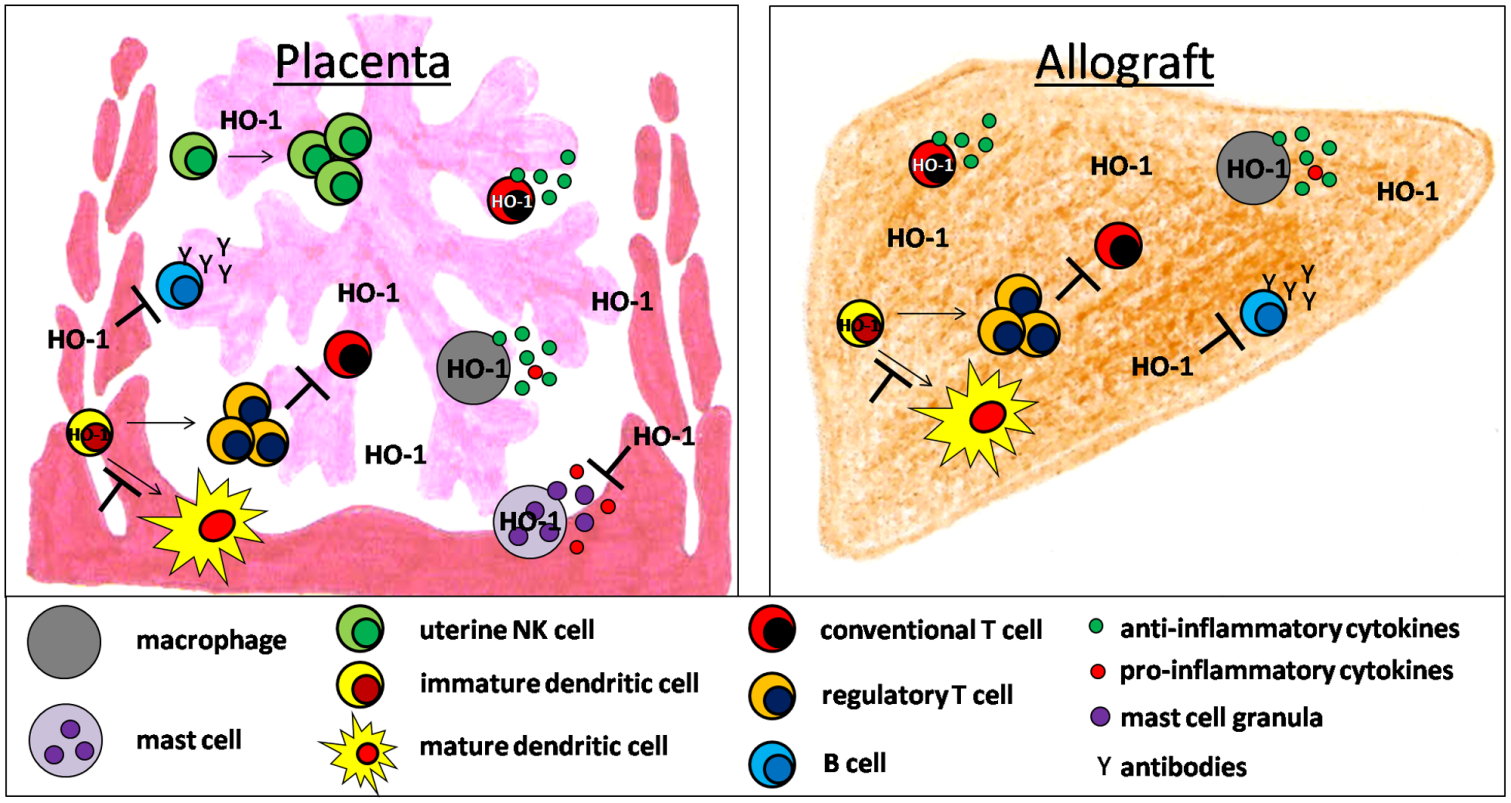
**Hypothetical scenario of HO-1-mediated effects on innate and adaptive immune cell populations supporting fetal and allotransplant tolerance**.

**FIGURE 2:**
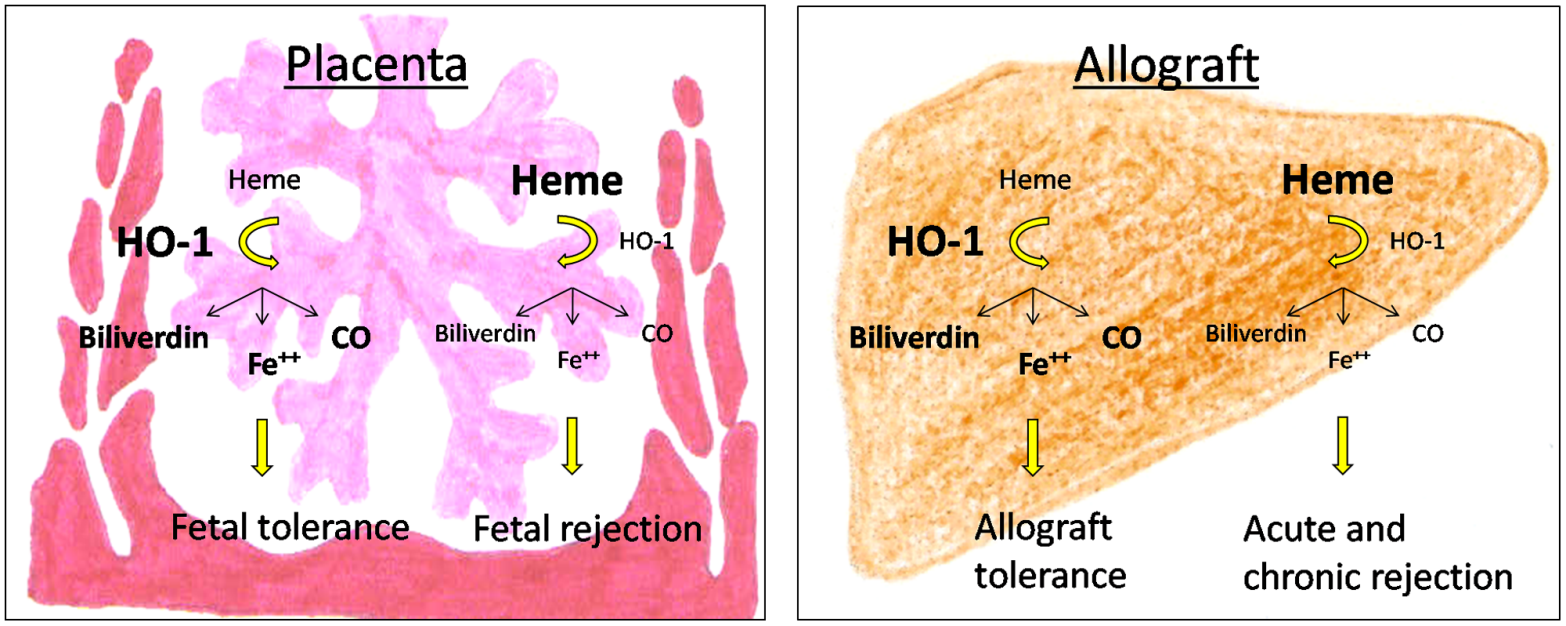
**Consequences of HO-1-deficiency for pregnancy outcome and allograft acceptance**.

There is accumulating evidence that HO-1 and its byproducts affect the development and function of a variety of immune cells during pregnancy and allotransplantation (**Figure 1**). Detrimental immune responses towards the foreign allo-antigens are modulated and/or suppressed allowing the survival of the fetus in the hostile uterine environment and the acceptance of the allograft by its recipient. However, HO-1 deficiency may result in fetal and allograft rejection (**Figure 2**). HO-1-based therapies may be promising strategies to circumvent both, fetal and allotransplant rejections. These approaches may include pharmacologic or gene therapy-mediated induction of HO-1 as well as the administration of CO. Treg known to be essential for fetal and allotransplant tolerance may hereby represent a major target of HO-1 modulation.

## Conflict of Interest Statement

The authors declare that the research was conducted in the absence of any commercial or financial relationships that could be construed as a potential conflict of interest.
